# Prescriptions

**Published:** 1896-05

**Authors:** 


DIABETES MELLITUS.

℞. Sodii salicylat........ ʒ iij,

Liq. potassii arsenitis. ʒ j.

Glycerinæ............ ℥ j.

Aquæ cinnamomi. .ad ℥ iij.

M. Sig.—A dessertspoonful three times daily.—J. C. WILSON.

DIARRHŒIA, CHILDREN.

℞. Olei ricini........... ʒ ij.

Pulv. acaciæ

Pulv. sacchari.. .aa ʒ ij.

Tinct. opii.......... ♏ xxj.

Aquæ cinnamomi .ad ℥ iv.

M. Sig.—A teaspoonful every three or four hours.—WEST.

DIARRHŒA ADULTS.

℞. Tinct. opii camph.
Spts. ætheris co.
Ext. valerianæ fld.aa ℥ ss.
Olei menthæ pip...gtt. xxx.
Spts. lavandulæ co.
..............q.s. ad ℥ iv.
M. Sig.—A teaspoonful every two or three hours.—WM. DARRACH.

GOUT.

℞. Colchicini ........... gr. j
Ext. Colocynth co..... ʒ ss.
Quiniæ sulph.......... ʒ iij.
M. Ft. massa et in pil. no. lx div. Sig.—One pill every four hours.—BARTHOLOW.

MANIA, PUERPERAL.

℞. Potassi bromidi........ ʒ ij.

Chloral hydratis.... . ʒ iv.

Syr. aurantii cort...... ℥ j.

Aquæ fœniculi.....ad ℥ vj.

M. Sig.—A tablespoonful every two hours.—QUAIN.

NEURALGIA.

℞. Menthol .........gr. xxiiss.
Cocaini muriatis.gr. viiss.
Chloral hydratis.gr. ivss.
Vaselini......... ʒ iiss.
Ft. ungt.
M. Sig.—Apply to the painful part and cover with a strip of court-plaster. (*Gor supra-orbitai neuralgia.*) GALEZOWSKI.

OPIUM HABIT.

℞. Tinct. nucis vomicæ...............gtt. xij.
Acidi phosphorici dil...............gtt. xx.
Syr. pruni virginianæ............. ℥ ss.
M. Sig.—To be taken twice daily.

PTYALISM (SALIVATION).

℞. Potassii chloratis..... ʒ j.
Aquæ................. ℥ vj.
M Sig.—Use as a mouth-wash and internally in teaspoonful doses four or five timesdaily. —STURGIS.

